# Learning to diagnose collaboratively: validating a simulation for medical students

**DOI:** 10.3205/zma001344

**Published:** 2020-09-15

**Authors:** Anika Radkowitsch, Martin R. Fischer, Ralf Schmidmaier, Frank Fischer

**Affiliations:** 1 Ludwig-Maximilians-Universität München, Munich Center of the Learning Sciences, München, Germany; 2 Ludwig-Maximilians-Universität München, Lehrstuhl für Empirische Pädagogik, Department Psychologie, München, Germany; 3 Ludwig-Maximilians-Universität München, LMU Klinikum, Institut für Didaktik und Ausbildungsforschung in der Medizin, München, Germany; 4 Ludwig-Maximilians-Universität München, LMU Klinikum, Medizinische Klinik und Poliklinik IV, München, Germany

**Keywords:** collaboration, simulation, collaborative diagnostic reasoning, validation

## Abstract

**Objectives: **Physicians with different professional backgrounds often diagnose a patients’ problem collaboratively. In this article, we first introduce a process model for collaborative diagnosing (CDR model), describe the development of a simulation used to empirically examine the facilitation of collaborative diagnostic reasoning. Based on a contemporary validity framework [1], we further suggest indicators for validity and collect initial evidence with respect to the scoring, generalization, extrapolation, and implication inferences to assess the validity of the simulation when used to assess effects of learning interventions.

**Method:** In a quasi-experimental study, we assessed objectivity and reliability of the simulation and compared medical students with low and advanced prior knowledge to practitioners with high prior knowledge with respect to their diagnostic accuracy, diagnostic efficiency, information sharing skills, and their intrinsic cognitive load. Additionally, we obtained authenticity ratings from practitioners with high prior knowledge.

**Results: **The results yielded satisfying initial evidence for the validity of the scoring and the extrapolation inferences as ratings are objective, and the simulation and the collaborative process is perceived as rather authentic. Additionally, participants on different levels of prior knowledge differ with respect to their diagnostic accuracy, diagnostic efficiency, information sharing skills, and their reported intrinsic cognitive load. With one exception (information sharing skills), the generalization inference seems to be valid as well.

**Conclusions: **We conclude that collecting validity evidence for the simulation was an important step towards a better interpretation of the simulation. We found that the simulation is an authentic and valid representation of the chosen collaborative situation and that the collected validity evidence offers sufficient evidence for an initial validation of the simulation. Nevertheless, the validation process highlighted some important gaps that need further consideration. We further conclude that applying a validation model to the context of empirical research is promising and encourage other researchers to follow the example.

## 1. Introduction

In their daily practice, physicians with different professional backgrounds often diagnose patients’ problems collaboratively. For example, an internist diagnosing a patient suffering from fever and shortness of breath might consult a radiologist to conduct a CT scan the results of which will be discussed afterwards. In those situations, physicians need to be able to diagnose individually, that means being able to gather and integrate case-specific information with the goal to reduce uncertainty to make a medical decision [2]. But they also need collaborative competences such as sharing of relevant information, negotiation, and coordination skills [3]. A recent review shows that collaborative diagnostic reasoning has been scarcely investigated empirically yet [4]. The available empirical literature demonstrates that physicians often have difficulties to diagnose collaboratively. For example, the quality of the distribution and exchange of information among team members [5] and the experience of team members [6] seem to be key predictors for the quality of collaborative diagnostic reasoning. Such difficulties in information sharing also could affect the quality of subsequent negotiation processes. For instance, if an internist fails to share differential diagnoses and the respective symptoms, the radiologist will have a much harder time to interpret and to discuss the radiologic findings. Offering instructional support to foster collaborative diagnostic reasoning and in particular information sharing, therefore, seems vital. Simulation-based learning is an established method to foster complex competences and its effectiveness has been meta-analytically examined for health professions [7] as well as across domains [8] although it seems that scaffolding beyond mere problem-solving is beneficial for learning [9]. We developed, therefore, a simulation with the goal to identify instructional conditions under which simulations effectively advance collaborative diagnostic reasoning. Importantly, training and assessment of competences presupposes evidence of its validity. We follow Kane’s [1] validity framework for the validation of instruments as suggested by Cook and Hatala [10]. 

In this paper, we want to collect initial evidence for validity of the simulation by constructing a validity argument for a simulation used to conduct experiments on the facilitation of collaborative diagnostic reasoning. For that, we first elaborate on a model of collaborative diagnostic reasoning and describe how simulations can be used to assess and facilitate complex competences. We further explain our validation approach based on Kane’s [1] framework as well as validity indicators that are based on theory. Afterwards, we shortly describe the development of our simulation which included several evaluation and revision cycles (cf. [11]). Finally, we present a validation study that was conducted to analyze the validity indicators and discuss the extent to which the results add to our validity argument. 

## 2. Collaborative diagnostic reasoning

**Figure 1:**
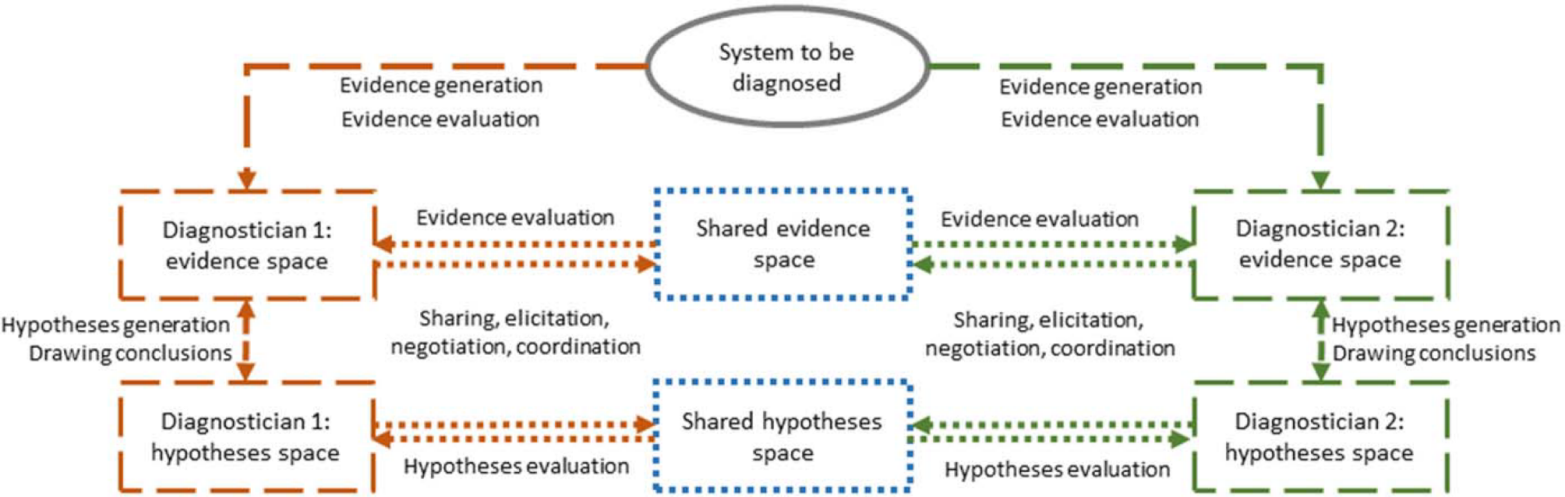
Model for collaborative diagnostic reasoning (CDR) adapted from Radkowitsch et al. [12]

Collaborative diagnostic reasoning means to accurately and efficiently diagnose a patient’s problem by generating and evaluating evidences and hypotheses that can be shared with, elicited from, or negotiated among collaborators [12]. In the medical and psychological literature, however, diagnosing has been largely conceptualized as individual competence and by using varying terms such as clinical or diagnostic reasoning, clinical decision-making, or clinical problem-solving (e.g., [13], [14]). When diagnosing individually, physicians generate and evaluate evidence based on patient information, weigh the evidence with respect to differential hypotheses and draw conclusions (i.e., make a medical decision) based on the diagnostic process [14], [15]. The quality of individual diagnostic activities is influenced by professional medical strategic and conceptual knowledge [16]. However, more than one diagnostician is often involved in diagnosing a patient or making treatment decisions. For example, in medical consultations a responsible physician calls in the expertise of another health-care professional. Another example are discussion rounds such as tumor boards in which physicians with different professional backgrounds exchange and discuss patient information. In both examples, diagnosticians have the joint goal to make the best clinical decision. When diagnosing collaboratively, the professional medical knowledge, the knowledge about the patient, and outcomes of diagnostic reasoning processes might differ between the diagnosticians. Therefore, collaborative activities are necessary in addition to the individual diagnostic activities to coordinate the individuals’ diagnostic processes. Based on the collaborative problem-solving framework by Liu and colleagues [3] and the scientific discovery as dual search (SDDS) model [17], Radkowitsch and colleagues [12] proposed a model for collaborative diagnostic reasoning (CRD model, see figure 1 (Fig. 1)) describing collaborative diagnostic processes with individual and collaborative diagnostic activities. These collaborative activities are sharing, elicitation, negotiation, and coordination. According to the CDR model, evidences and hypotheses generated and evaluated during diagnostic processes are kept in individual diagnostic spaces (dashed lines and boxes). All evidences and hypotheses that are available to all collaborators are represented in shared diagnostic spaces (dotted boxes). For evidences and hypotheses to become part of a shared diagnostic space, the diagnosticians need to conduct the proposed collaborative activities (dotted lines). For example, an internist diagnosing a patient suffering from fever and shortness of breath might generate the hypothesis of pneumonia. In order to reduce the uncertainty of this hypotheses, the internist consults a radiologist to perform a radiologic test. The quality and relevance of the information that the internist shares with the radiologist may influence the hypotheses generated and the conclusions drawn by the radiologist and further affect, which information is shared, negotiated or elicited by the radiologist. In turn, the evidences and hypotheses shared, elicited, or negotiated by the radiologist may influence the internist’s individual diagnostic process. Hence, the proposed collaborative activities are considered important for the quality of medical decisions. Based on models and findings on team cognition, we assume that the quality of collaborative activities is influenced by the team members’ meta-knowledge [18], [19]. By meta-knowledge we mean the knowledge a team member holds about the other team members’ roles, their knowledge, and their task. Meta-knowledge has been shown to particularly influence collaborative activities of collaborators (e.g., [20]). Among collaborative activities, information sharing has received particular attention. Sharing or rather the lack of sharing can affect the accuracy of the diagnoses, but at the same time diagnosticians often fail to share relevant information with others [5], [21]. 

## 3. Conducting research on advancing collaborative diagnostic reasoning with simulations

Simulations are an established method to foster competences in medical education as well as in other educational contexts such as teacher trainings [22], pilot trainings [23], or military trainings [24]. In all these contexts, the application of knowledge is a crucial part of professional practice [e.g., [25]. Simulations allow to practice the application of knowledge in a risk-free environment [26]. More importantly, however, simulations allow for the deliberate practice [27] of particularly difficult or complex subtasks. That means that within simulations, learners can repeatedly solve (sub-)tasks that they are yet not able to complete. Research on the deliberate practice has shown that this type of practice is particularly crucial during the development of professional expertise [27]. Besides, the application of knowledge in complex domains such as medicine can be overwhelming for learners. To facilitate learning, the complexity of these situation can be reduced in simulations and thereby offer a tradeoff between an approximation-of-practice and authentic representations of real-world situations [28]. Research on the effectiveness of simulations shows positive effects on cognitive, behavioral, and affective learning outcomes in medicine as well as in other domains [7], [8], [29]. However, a recent review shows that to advance diagnostic competences, the provision of additional instructional support beyond the opportunity to solve problems is beneficial [9]. We propose a research agenda to investigate conditions under which diagnostic competences are effectively advanced when learning with simulations [2]. For empirical laboratory research on complex competences it is necessary to focus on empirically measurable aspects. Hence, we focus on information sharing as subskill of collaborative diagnostic reasoning. 

When conducting research on the effectiveness of different instructional means, educational research typically uses controlled experiments. That means that two or more groups of learners receive different types of support in an intervention phase. By using unsupported pre- and post-tests, the learning gain of the different groups of learners is assessed [e.g., [30]. The average performance of groups is then compared to identify the effects of the intervention. To realize the proposed research agenda [2], we developed a simulation that will be used in experiments to facilitate but also to assess collaborative diagnostic reasoning, in particular the sharing of information during diagnosing. During the intervention, learners will receive different versions of the simulation. During the pre- and posttest, the simulation will be used to assess the competence levels of groups of learners. Hence, it is an important prerequisite that the simulation differentiates between different competence levels, as well as that the simulation is suitable for the competence level of the targeted group. Using simulations for the assessment of competences is a common approach in medical education [31]. For example, simulations are used to assess procedural skills such as conducting rectal examinations [32], medical communication skills [33], or diagnostic reasoning [34]. When using simulations to assess competences, it is highly relevant that the simulation consists of authentic representations of real-world situations in which the respective competences is typically used [31], [35]. For assessing diagnostic reasoning skills, simulations usually present patient cases for which learners need to come up with the most likely diagnosis [31]. A systematic review on simulations shows that the evaluation of simulations with respect to their validity as assessment tool lacks thoroughness [36]. Therefore, in the present paper we seek to examine whether the simulation developed to realize our research agenda is a valid instrument for the assessment of between group differences of competence levels. 

## 4. Validating a simulation of collaborative diagnostic reasoning: constructing a validity argument

In his validity framework, Kane [1] describes validation as the process to collect and to evaluate validity evidence to judge the appropriateness of interpretations of the results of the assessment. Four typical inferences are drawn when concluding from a test score to a real score which need critical examination with respect to their validity: scoring, generalization, extrapolation, and implications. Each of these inferences are typically based on implicit assumptions that need to be considered during a validation process [10], [37]. In this paper, we explicate these assumptions for the simulation-based assessment of collaborative diagnostic reasoning that has the goal to identify conditions under which collaborative diagnostic reasoning can be effectively facilitated. All considered assumptions as well as their warrants are listed in table 1 (Tab. 1). The first inference, scoring, refers to matching an observation to a single score [38]. For example, in our simulation a medical student proposes a diagnosis for a patient case which is then scored by the experimenter. A valid scoring procedure requires the observations to be correctly transformed into a consistent score and that raters of the accuracy of the final diagnoses show reliable ratings as indicated by high inter-rater agreements (assumption 1.1). The second inference, generalization, refers to generalizing the single score to a test score [38]. In our simulation, we generalize from the information sharing skills shown in one simulated patient case to the information sharing skills shown in several other simulated patient cases. A valid generalization inference is shown, if scores on a single performance (e.g., a final diagnosis of one patient) aligns with an overall score (e.g., all final diagnoses given during the test setting). Hence, high internal consistency of the measures are indicators for plausible extrapolation inferences (assumption 2.1). Extrapolation refers to generalizing from the test score to the real performance [38]. In our simulation, we would hope that medical students who are better in collaborative diagnostic reasoning in our simulation would also be better in collaborative diagnostic reasoning when working with real patients and colleagues. Hence, validity evidence should ideally show that the collaborative diagnostic reasoning of groups of learners shown within our simulations is representative for their collaborative diagnostic reasoning outside the simulation. To ensure that, we propose several validity indicators: First of all, it would be strong evidence for a valid extrapolation inference if experienced practitioners from the field rated the simulation as authentic (assumption 3.1) [35]. We consider experienced practitioners able to judge whether the simulated setting represents real life practices. Secondly, a valid assessment requires that medical practitioners and medical students with high prior knowledge show better test performance (i.e., more accurate and more efficient diagnostic performance) compared to medical students with low prior knowledge (assumption 3.2). The assumption is that on average those showing higher performance in real life settings on average also show higher performance within the simulation. A third validity indicator for the extrapolation inference are differences between persons with different levels of prior knowledge with respect to cognitive load. The cognitive load theory assumes that learning imposes different kinds of cognitive load on learners. Particularly, the intrinsic cognitive load which is caused by the complexity of the learning material should be lower for people with high prior knowledge compared to less knowledgeable medical students [39]. With higher prior knowledge, the learning material becomes less complex as the material is better cognitively organized and, therefore, imposes less intrinsic load (assumption 3.3). Importantly, to assess the effectiveness of different kinds of simulations, we compare groups of learners rather than individuals. That means that all decisions will be based on group means rather than individual test results. Therefore, a further assumption is that differences between groups of learners result from the intervention and not from random or systematic prior differences between groups (assumption 3.4). Therefore, it is important to use an experimental approach. The final inference, implications, refers to the conclusions drawn, and decisions made based on the test results [1], [10], [38]. Hence, the final assumption is that the resulting data can be used to draw inferences on the effectiveness of different kinds of simulations (assumption 4.1). If the prior assumptions were met, then the implications drawn from the results would be valid. 

Considering the intended use of the instrument to be validated is important for the construction of a validity argument as this helps to prioritize the evidence [10]. The intended use of the simulation described in this paper is to assess collaborative diagnostic reasoning of groups of learners in experimental studies. Although every described validity evidence is considered important for the construction of the validity argument, some of the evidences are considered crucial. For our intended use, we argue that particularly the identification of different levels of competence among participants with different levels of prior knowledge would offer the most important validity evidence as this evidence is closest to the final use of the simulation. Although due to content specificity of diagnostic skills, it seems hard to achieve reliable measures in medicine [13], [31], it is particularly important to have coherent measures that allow generalizing from one item to another as this would offer evidence that the same skill is assessed in different items. 

## 5. Research questions of the validation study

Based on the validity framework and the validity indicators described above, we conducted a validation study to answer the following research questions:

1. Scoring: To what extent are the measures of collaborative diagnostic reasoning objective?
2. Generalization: To what extent are the measures of collaborative diagnostic reasoning consistent?
3. Extrapolation:
4.
  1.
    1. To what extent do medical practitioners perceive the simulation as authentic?
  2.
    1. To what extent do groups with different levels of prior knowledge differ with respect to a) their collaborative diagnostic reasoning (information sharing skills, diagnostic efficiency, and diagnostic accuracy) within the simulation and b) to the reported intrinsic cognitive load?

## 6. Method

### 6.1. Development of the simulation to assess collaborative diagnostic reasoning 

**Figure 2:**
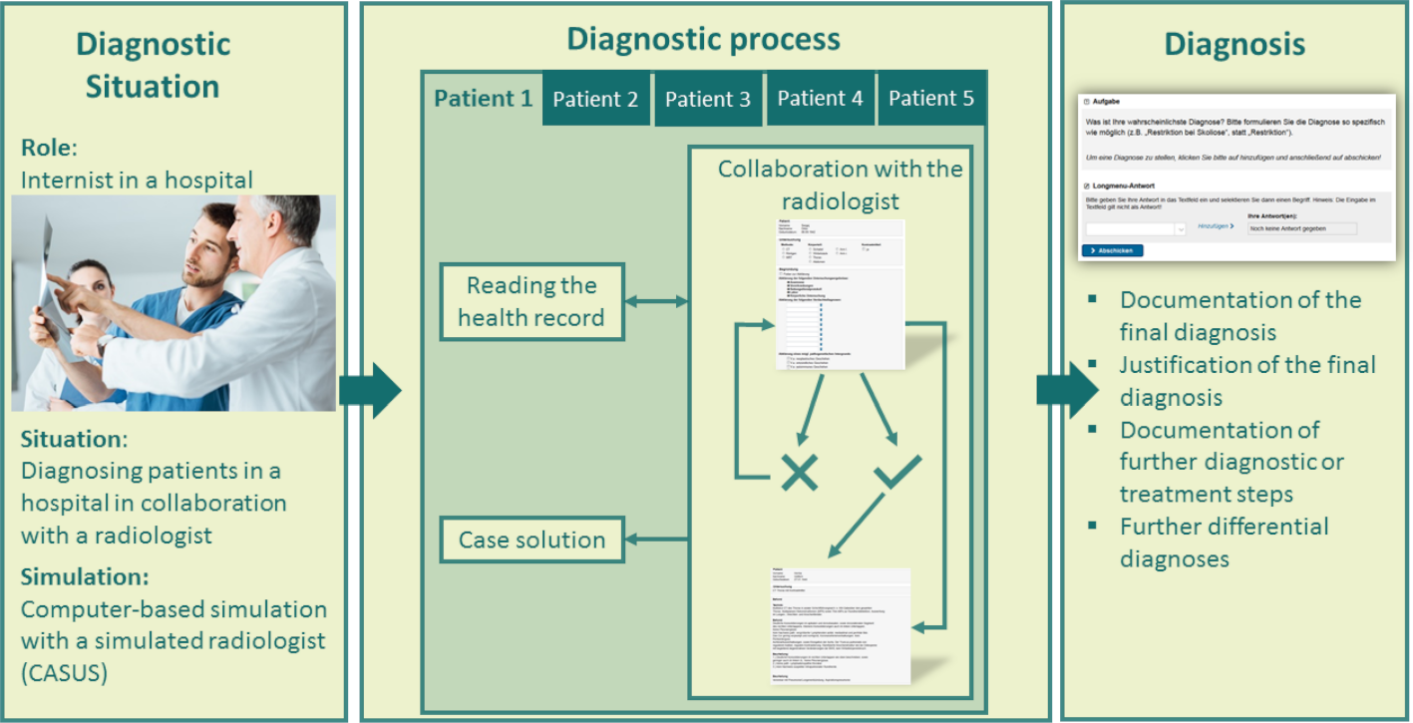
Schematic representation of the simulation

Our goal is to develop a tool for the assessment of the specific subskills of collaborative diagnostic reasoning as defined above. We chose a simulation-based approach to assess collaborative diagnostic reasoning [7], [8]. As described above, the construct of collaborative diagnostic reasoning is rather broad and can be assessed in a broad range of contexts. For example, different physicians such as internists, surgeons, or gynecologists could collaborate with nurses or other health-related professionals. We assume that the context of collaboration (such as the meta-knowledge about the collaborators’ profession) influences collaborative diagnostic processes. We, therefore, decided to narrow down the simulated context to a situation that is relevant in real-world practices and particularly difficult for learners. Hence, we defined the simulated context as a collaborative situation between internists and radiologists based on practitioners’ experiences. Interviews with seven practitioners from both disciplines were conducted to identify a specific situation that is considered as being problematic frequently. The interviews yielded that the main problem is unspecific test requests, that is unprecise justifications for the test (e.g., missing relevant patient information) and a lack of clustering of patient information. As a consequence, we decided to focus on information sharing during the request of a radiologic examination as an important and specific aspect of collaborative diagnostic reasoning. Next, we decided to use a computer-based simulation and chose the case-based learning platform CASUS (https://www.instruct.eu/). Computer-based simulations have several advantages compared to other types of simulations such as standardized patients (e.g., [33]). First, the use of the simulation is extremely economical once the material is developed as several participants can interact with the simulation at the same time and, for example, no actors are needed. Secondly, web-based simulations are easily accessible for participants and, hence, time and place restrictions are low. Thirdly, all case material as well as instructions are standardized and, therefore, do not confound the assessment. To develop the simulation, paper prototypes of the scenario and patient cases were constructed and evaluated by an expert committee from medicine, software development, and psychology. Whereas internists, radiologists, and a general practitioner developed the case material for ten patient cases, a software developer programed the simulation. The case material was then evaluated and revised in a one-day expert-workshop, with focus on the case structure, the most plausible solution, as well as the sample solution. Finally, the simulation was implemented on the CASUS learning platform (see figure 2 (Fig. 2)). 

In a pilot study, the simulation with one patient case was presented to eight medical students (*M*_age_=24.5, *SD*_Age_=3.9; *M*_Semester_=7.6, *SD*_Semester_=1.2) to evaluate the user experience of the simulation (UEQ; [40]). Results indicated high values on the subscales attractiveness, perspicuity, stimulation, and novelty, but rather low values on the subscale dependability. To increase the perceived control for participants, a fiction contract containing information about the simulated scenario and the role learners are expected to take up as well as a technical familiarization giving detailed instructions on how to handle the simulation were developed. After having read the fiction contract and the familiarization, participants start the first simulated patient case. Participants first receive a patient file that they scan for symptoms and findings in the role of an internist. The patient file consists of a short patient presentation, medical history, a description of the physical examination, as well as the most important laboratory values. Afterwards, learners request a radiologic test from a simulated radiologist. For that, they are asked to fill in a request form by choosing among 42 different combinations of methods and body parts and by sharing patient information or differential diagnoses that are considered relevant for the radiologist. Only learners who appropriately justified their request (i.e., show high information sharing skills) receive a description of the radiologic findings, and, if provided by the learner, an evaluation of a specific differential diagnose from the simulated radiologist. We decided beforehand with radiologists which information is needed to justify a specific radiologic test. After having read the radiologic result, medical students can ask questions about the radiologic findings, share further information, or request further examinations. To solve the patient case, participants suggest a diagnosis and back it up with justifying findings and suggest further differential diagnoses and treatment or diagnostic measures. For a more detailed description of the simulation and the process of development, see [12]. In sum, in our simulation medical students are supposed to gather and integrate information from a patient file, and to collaboratively generate radiologic evidence by sharing relevant patient information with the radiologist. By that the medical student elicits relevant information from the radiologist, which they then integrate into prior information to arrive at a final diagnosis. Bearing in mind our definition of collaborative diagnostic reasoning, the simulation allows us to separately assess and facilitate both, collaborative diagnostic reasoning (i.e., information sharing) as well as individual diagnostic reasoning (i.e., the final diagnosis). 

#### 6.2. Sample and design

A quasi-experimental study with a one-factorial design consisting of three levels (low vs advanced vs high prior knowledge level) was conducted. We defined medical students between the 5^th^ and 8^th^ semester (*N*=45, *N*_female_=31) of a total of 12 semesters as low prior knowledge (PK) (*M*_PK_=6.4 semesters, *SD*_PK_=0.7) as they had only few courses on internal medicine and radiology according to their study plan. Medical students from the 9^t^h semester and above (*N*=28, *N*_female_=19) were categorized as advanced prior knowledge (*M*_PK_=11.5 semesters, *SD*_PK_=1.9) as they already participated in courses for internal medicine and radiology according to their study plan. Internists and residents for Internal Medicine after completion of the 3 years of common trunk (*N*=25, *N*_female_=11) were categorized as high prior knowledge (*M*_PK_=13.6 years, *SD*_PK_=10.5) as they are expected to have practical experience. 

#### 6.3. Procedure

The study was conducted as a laboratory study with a maximum of eight participants at a time. All participants consecutively worked individually on five computer-based patient cases as described above for as long as they wanted. The participants were asked to work efficiently. After the second and the fifth case, participants completed a test measuring perceived authenticity as well as intrinsic cognitive load. Afterwards, participants were debriefed and thanked for their participation with 25€.

#### 6.4. Measures

Within the simulation, we obtained three measures to assess the collaborative diagnostic reasoning: diagnostic accuracy, diagnostic efficiency, and information sharing skills. We used Likert-scaled items to assess the perceived authenticity of the simulation as well as the perceived intrinsic cognitive load (see table 2 (Tab. 2)). 

##### Diagnostic accuracy

The solution of the patient case (i.e., the suggested final diagnosis), differential diagnoses, and further necessary diagnostic or treatment steps were used to score the diagnostic accuracy. Depending on how specific the given diagnosis was, participants received 0, 0.5 or 1 point for each diagnosis and up to one additional point each for the quality of the differential diagnoses and the quality of the indicated further steps. Points were given based on the sample solution that was developed in the expert workshop. The mean diagnostic accuracy across the five patient cases (ranging from 0 to 3) was calculated for each participant. 

##### Diagnostic efficiency

The diagnostic accuracy weighted by the time needed to solve a single patient case indicated the diagnostic efficiency. The mean diagnostic efficiency across the five patient cases was calculated for each participant.

##### Information sharing skills

The information sharing skills were operationalized as the inverted proportion of requests rejected by the simulated radiologist due to insufficient justification per case. Whether a justification is perceived as sufficient or insufficient by the simulated radiologist was defined beforehand in collaboration with expert radiologists based on how relevant information is for a radiologist to conduct a radiologic test. For this measure, values were obtained directly via the logfiles. The mean score of all five patient cases (ranging from 0 to 1) was calculated for each participant. A mean score of 1 means that all requests in all patient cases were accepted by the radiologist.

##### Perceived authenticity

The perceived authenticity was assessed with three items each with respect to the overall simulation and with respect to the collaborative process [41] on a 5-point Likert scale ranging from 1 (does not apply) to 5 (does apply). The perceived authenticity of the simulation as well as the authenticity of the collaborative process was assessed twice. An example item for authenticity is “I perceive the [simulation] / [the collaboration with the radiologist] as authentic”. 

##### Intrinsic cognitive load

Intrinsic cognitive load was assessed with one item on a 5-point Likert scale ranging from 1 (very easy) to 5 (very difficult) [42]. The item text was “How easy or difficult do you find the collaboration with a radiologist at the moment?”.

#### 6.5. Statistical analyses

To answer research question 1, we obtained the intraclass correlation (ICC) based on a two-way random effects model with absolute agreement for the main diagnoses, the differential diagnoses, and the indicated further steps. For that, two raters independently coded 20% of the cases. 

To address research question 2, we calculated the internal consistency measure Cronbach’s alpha with respect to the diagnostic efficiency, to the information sharing skills, and to the diagnostic accuracy. 

To answer research question 3.1., we calculated the mean of both measurement times and contrasted it to a threshold of 3.0 using a one-sample t-test. The means above the threshold indicate that participants with high levels of prior knowledge on average rate the overall simulation and the collaborative process as rather authentic or authentic. 

To address research question 3.2., we conducted ANOVAs and Bonferroni post-hoc tests with the independent variable prior knowledge and the dependent variables diagnostic accuracy, diagnostic efficiency, information sharing skill, as well as intrinsic cognitive load. If preconditions for calculating an ANOVA were not met, we conducted the non-parametric Kruskal-Wallis-Test and Wilcoxon post-hoc tests instead. Confidence intervals are calculated with bootstrapping. 

## 7. Results of the validation study

### Scoring

With respect to the first research question, we obtained high values for all three variables: The interrater agreement for the quality of the final diagnoses and for the further indicated steps was ICC=1. For the differential diagnoses, the interrater agreement was ICC=0.94. This indicates that raters objectively scored the observations during the simulation. 

#### Generalization

With respect to research question 2, analyses yielded a Cronbach’s alpha of .66 for the diagnostic accuracy, a Cronbach’s alpha of .53 for the diagnostic efficiency, and a Cronbach’s alpha of .33 for the information sharing skills. This indicates that the evidence for the generalization inference being valid is acceptable for the diagnostic accuracy and the diagnostic efficiency but limited for the information sharing skills. 

#### Extrapolation

**Figure 3:**
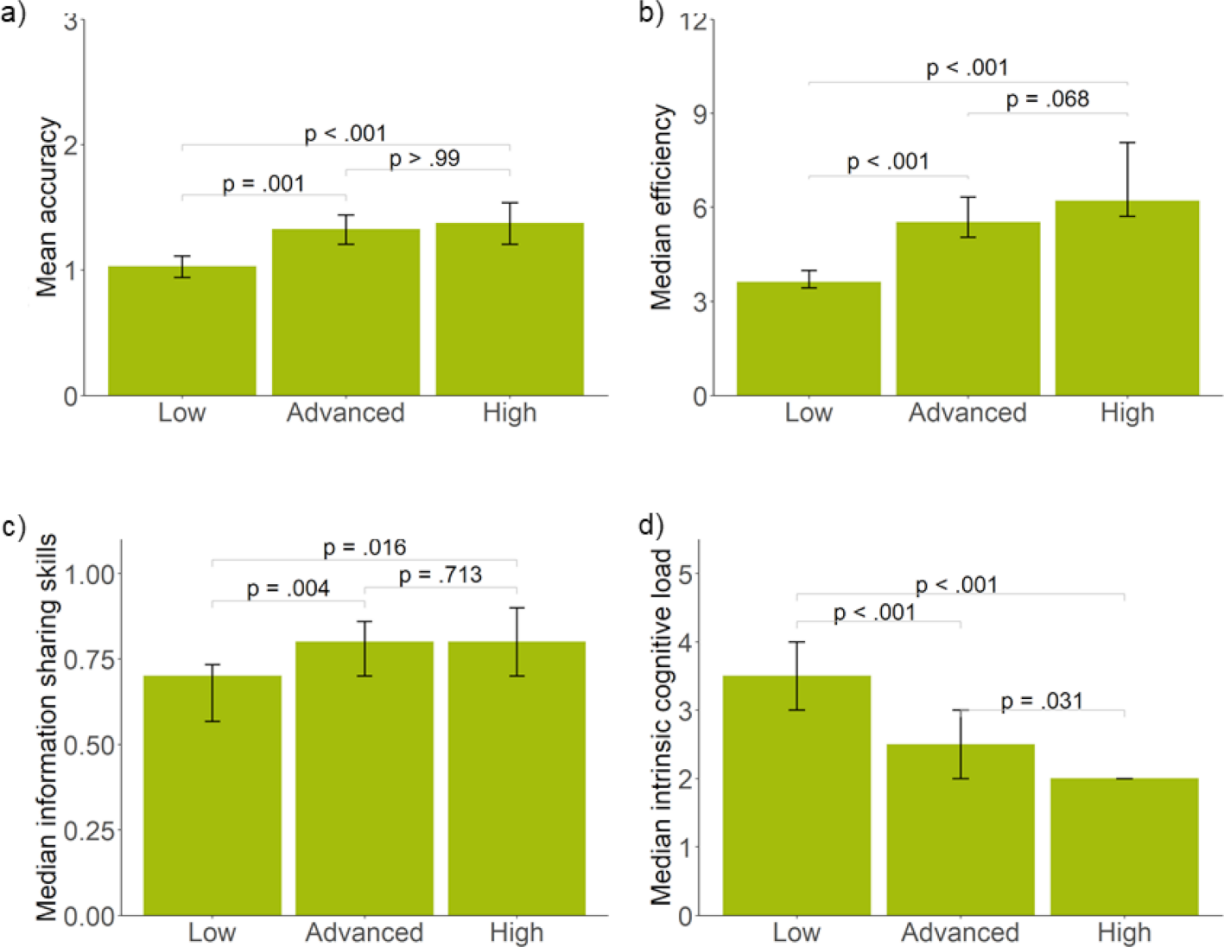
Differences of prior knowledge groups with respect to a) diagnostic accuracy, b) diagnostic efficiency, c) information sharing skill, and d) intrinsic cognitive load. Error bars indicate 95% Confidence Intervals.

With respect to research question 3.1., participants with high prior knowledge rated the perceived authenticity of the overall simulation as *M*=3.89 (*SD*=0.91) and the authenticity of the simulated collaborative process as *M*=3.57 (*SD*=0.91). Both authenticity ratings are significantly above the threshold of 3 (*t*(24)=4.9, *p*<.01 and *t*(24)=3.14, *p*<.01). This indicates that, on average, practitioners with high levels of prior knowledge perceive the simulation as rather authentic or authentic. Concerning research question 3.2., see table 3 (Tab. 3) for the descriptive statistics and figure 3 (Fig. 3), a-d for between-group comparisons. The results show that the prior knowledge groups differ significantly with respect to the diagnostic accuracy (*F*(2,95)=11.62, *p*<.001, *η^2^*=0.20). The high and advanced prior knowledge group show significantly higher accuracy than the low prior knowledge group but are not significantly different from each other. However, we found solution rates of up to 0.94 (i.e., the correctness of the final diagnosis) for three of the five patient cases indicating ceiling effects for the final diagnoses. The prior knowledge groups also differ significantly with respect to the diagnostic efficiency (*χ^2^*(2)=34.29, p<.001, *η^2^*=0.34) and with respect to the information sharing skills (*χ^2^*(2)=12.48, p<.002, *η^2^*=0.11). For both outcomes, the high and advanced prior knowledge groups again outperform the low prior knowledge group but do not differ significantly from each other. The prior knowledge groups further differ with respect to the reported intrinsic cognitive load (*χ^2^*(2)=38.25, p<.001, *η^2^*=0.38). The high prior knowledge group reported the lowest intrinsic cognitive load, followed by the advanced, and the low prior knowledge groups. All comparisons are statistically significant. 

## 8. Discussion

The objective of this study was to collect initial validity evidence for the simulation we developed to conduct further experimental research on facilitating collaborative diagnostic reasoning in medical education. The validation of the simulation was based on a theoretical model describing collaborative diagnostic processes (CDR model; [12]). The simulation focusses on one of the proposed collaborative activities, namely information sharing. The CDR model suggests that which information is shared by one diagnostician influences the diagnostic processes of another diagnostician. In case of the simulation, which information is shared by a learner in the role of an internist influences whether a radiologist conducts a radiologic test and how it is interpreted. An argument for initial validity was constructed by applying Kane’s [1] validity framework to the context of experimental research based on a simulation. The underlying assumptions were made explicit and supported by warrants (see table 1 (Tab. 1)). However, the strength of these warrants varies between inferences. We were able to show quite clearly that the single observations within the simulation can be assessed objectively as all materials were developed and evaluated by expert committees from different disciplines, and some of the variable scores are generated automatically (scoring). This reduces human errors during the transformation of the observation to a single score. For the variables where coding was necessary, inter-rater reliability was high. We conclude that no further evidence for the validity of the scoring procedure is necessary. Further, we found satisfying validity evidence for the question whether the results of the simulation can be transferred to real-world scenarios by comparing participants with different prior knowledge with respect to their performance and their indicated cognitive load in the simulation (extrapolation). We find that medical students and practitioners with high levels of prior knowledge indeed show higher information sharing skills than medical students with low levels of prior knowledge. This indicates that the simulation enables differentiating between levels of competence of different groups which is the intended use of the simulation. However, there is one exception. We found rather high solution rates for the patient cases, even with students on low levels of prior knowledge, indicating ceiling effects for the case solution included in the measures diagnostic accuracy and diagnostic efficiency. Higher case difficulty would allow to better distinguish between different levels of the competences under consideration which is why case difficulty was increased by adding further distracting information. Nevertheless, it is a recurrent finding in medical education that intermediates and experts do not differ in the accuracy of the diagnoses, but rather in the efficiency with which they come up with the correct solution [31]. An explanation for this effect is that the knowledge of experts is better organized (i.e., encapsulation of knowledge) compared to the knowledge of intermediates. This superior organization of knowledge enables experts to more efficiently come to a correct diagnosis [43]. This pattern of effects is illustrated in our data as the difference between intermediates and experts is descriptively larger for diagnostic efficiency than for diagnostic accuracy. Furthermore, the simulation was rated as rather authentic by practitioners from the field. Ultimately, when conducting experiments with the simulation to compare learning gains of groups of learners, it is of prime importance to additionally rule out prior differences between groups as confounding factors. This could be achieved by randomly distributing learners to experimental groups and by controlling for prior knowledge. Assuming that the simulation is used in randomized experiments, the validation study yielded satisfying evidence for the extrapolation inference. The weakest evidence was found for the assumption that scores from a single observation can be reliably summarized to an overall score (generalization). For two of the three variables of interest (diagnostic efficiency and diagnostic accuracy), the validity evidence is acceptable. For the information sharing skills, we obtained only low internal consistency indicating that across patient cases, learners show varying levels of information sharing quality. One explanation for the generally rather low value might be the small number of observations as the likelihood of higher reliability values increases with the number of observations. Generally, low consistency across different patients is a well-known problem in medical education and is also known as content specificity [13]. That means that the diagnostic accuracy between patient cases correlates poorly (0.1-0.3) [13]. That the consistency across patient cases is particularly low for collaborative diagnostic activities such as information sharing might be explained by the CDR model: Whereas individual diagnostic processes are influenced by medical knowledge, collaborative diagnostic reasoning is further influenced by the professional collaboration knowledge (e.g. meta-knowledge). For example, a student might know which information to share for a patient suffering pneumonia, but not for a patient suffering lung cancer. Hence, the measure for information sharing skill might be affected by both, professional medical content knowledge and professional meta-knowledge about the collaboration partners’ discipline. Hence, the presented evidence for the generalization inference, particularly for information sharing skills, of our simulation gives rather limited support for the validity which is why further evidence is necessary. 

### 8.1. Limitations

Of course, the present study is not without limitations that must be considered when interpreting its findings. First of all, the simulation is meant to represent collaborative diagnostic reasoning, however, we focus on a very specific subskill which is the sharing of information in diagnostic situations. This is a narrow focus and the results will not easily generalize to other subskills such as negotiation of differential diagnoses. However, we consider the subskill sharing as a particularly important part of collaborative diagnostic reasoning as prior literature has shown how important and how error-prone the sharing of relevant information is for the field of medicine (e.g., [5], [21]). Similar findings have also been reported in other fields (e.g., [20], [44]). The simulation will be used to scaffold the learning of sharing processes and we are convinced that our findings will be of use in other diagnostic situations in which sharing among diagnosticians is necessary as well. 

Additionally, our validity argument is based to a large extent on a comparison between experts and novices. Such comparisons have been criticized as novices and experts differ in several variables which are oftentimes unrelated to the construct under investigation such as the probability of having grey hair ("grey hair index", [45], p. 830). However, we do not intend to argue that the expert-novice comparison shows that we’re actually measuring the construct of interest. Instead, we argue that the expert-novice comparison shows that we are able to measure competence differences between groups using the simulation. Also, the intended use of the simulation is not to make judgements about individual competences of learners but rather to compare learning gains of groups to make judgements about the simulation’s effectiveness under different instructional conditions. Therefore, we consider the results of comparisons between different levels of prior knowledge as a meaningful contribution to our validation argument. 

## 9. Conclusion

In this article, we presented the collection of initial validity evidences for the simulation which we developed to investigate the facilitation of collaborative diagnostic reasoning – and more particularly information sharing – with simulations. Our validation process allows concluding that the simulation that was developed based on theory is indeed authentic enough with respect to both diagnostic process and collaboration. Importantly, more advanced students and practitioners are more efficient than students in earlier phases of their studies and experience less intrinsic cognitive load. More knowledgeable learners are also better able to interact successfully with the simulated radiologist. Thus, we were able to find initial validity evidence that the simulation can be used to assess whether interventions differ in their impact on the learning of collaborative diagnostic reasoning. With respect to the assessment of the information sharing skills as subcomponent of the collaborative diagnostic reasoning there is, however, a need for improvement concerning the reliability. As the reliability of assessments is considered one of the most important evidence components, this is still an important gap in the validity argument. Refining the measurement and increasing the number of observations might help to close this gap. 

Collecting validity evidence about simulations for diagnostic reasoning still seems uncommon [36]. Yet, the construction of a validity argument helped us to understand the strength and weaknesses of the simulation for its intended use. This is an important step and will help us to interpret the results of planned experiments. Besides some gaps in the validity argument that will be addressed further, the simulation is a solid instrument to empirically examine the advancement of collaborative diagnostic reasoning of medical students. 

## Funding

The research presented in this contribution was funded by a grant of the Deutsche Forschungsgemeinschaft (DFG) to Frank Fischer, Martin R. Fischer and Ralf Schmidmaier (FI 792/11-1).

## Competing interests

The authors declare that they have no competing interests. 

## Figures and Tables

**Table 1:**
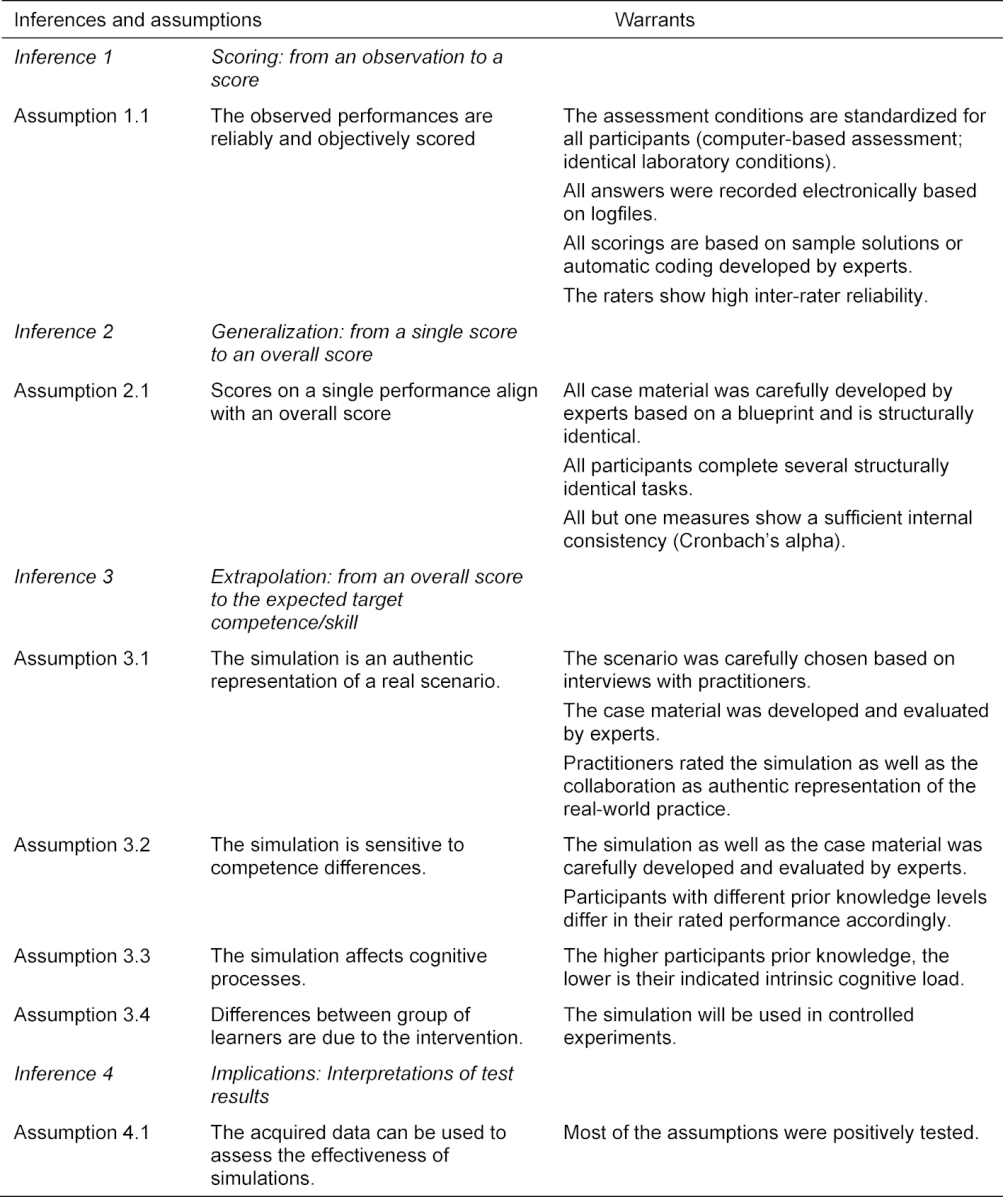
Inferences, assumptions, and warrants for the development of the argument of validity

**Table 2:**
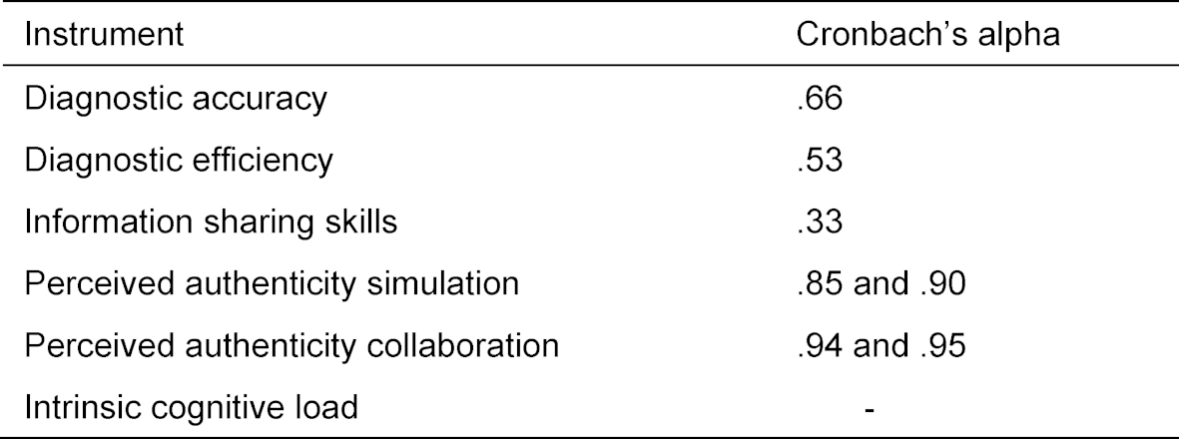
Internal consistencies for all instruments

**Table 3:**
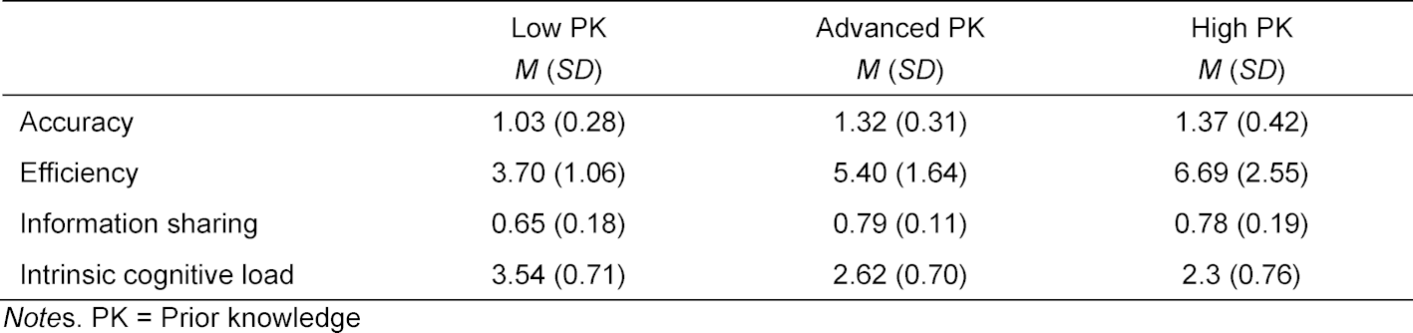
Means and standard deviations per variable and group.
